# Soft palate angle and basihyoid depth increase with tongue size and with body condition score in horses

**DOI:** 10.1111/evj.14445

**Published:** 2025-01-02

**Authors:** Alison M. Talbot, Hannah Shanks‐Boon, Christopher M. Baldwin, Harriet Barnes, Thomas W. Maddox

**Affiliations:** ^1^ University of Liverpool, Leahurst Campus Neston UK; ^2^ Present address: Bourton Vale Equine Clinic Gloucestershire UK

**Keywords:** basihyoid, body condition score, horse, obesity, soft palate, tongue

## Abstract

**Background:**

Obesity has been associated with human obstructive sleep apnoea and canine brachycephalic obstructive airway syndrome. The effect of body condition score (BCS) on structures of the oropharynx, nasopharynx and upper airway of the horse has not been investigated.

**Objectives:**

To investigate the effect of BCS on tongue measurements, soft palate angle and basihyoid depth in horses.

**Study design:**

Retrospective, analytical, cross‐sectional.

**Methods:**

Computed tomographic (CT) images of the head of 58 horses were assessed. DICOM viewing software was used to measure head length, basihyoid‐skin depth, soft palate angle (SPA), midline tongue area, dorsoventral height (DVH) of the tongue in two locations and head angle. BCS were assigned during CT examinations. Associations between measurements were tested and following initial calculations, further associations with tongue measurements as a ratio of head length were assessed.

**Results:**

For initial measurements, 44 horses met the inclusion criteria. Addition of head length ratios to tongue measurements resulted in 24 of 44 horses meeting the inclusion criteria for the second set of calculations. Increased BCS led to an increased mean SPA (mean difference = 2.56; *p* = 0.02) and increased median basihyoid depth (mean difference = 0.246 cm; *p* = 0.006). Following adjustments made for the effect of head length on tongue measures, significant correlation was identified between SPA and tongue area (Spearman's *r* = 0.544; *p* = 0.007); SPA and DVH of the tongue at the level of the hard palate (Spearman's *r* = 0.562; *p* = 0.004) and SPA and DVH of the tongue at the lingual process of the basihyoid bone (Spearman's *r* = 0.690; *p* < 0.001). No significant correlation was identified between variables and sex.

**Main limitations:**

The sample size was small and the effect of breed on measures was not studied. Measurements were acquired on a single sagittal CT plane. The investigator collecting CT measures was not blinded to BCS. All horses were sedated for the CT procedure which may have affected measures obtained.

**Conclusions:**

Increased BCS increases SPA and basihyoid bone depth. Increases in tongue size measurements increase SPA. Results from this study warrant further investigation into the clinical significance of the effects of BCS on the upper airways of the horse.

## 1 INTRODUCTION

Obesity is a growing concern in equids in the developed world and has been implicated as a risk factor in the development of multiple diseases including laminitis, equine metabolic syndrome, behavioural changes, reproductive problems and lower airway disease.^1^, ^2^, ^3^, ^4^, ^5^, ^6^ Anecdotally, overweight horses are often observed to make an increased inspiratory and expiratory noise at exercise, often referred to in lay terms as ‘thick winded’; however, objective studies as to the site of possible obstruction in these horses are lacking.

In humans, obesity has been linked to obstructive sleep apnoea (OSA).^7^, ^8^ In dog breeds such as Pugs and Bulldogs obesity results in increased risk of brachycephalic obstructive airway syndrome (BOAS), with many clinical consequences.^9^ In addition to overall body mass index (BMI), recent studies in humans and dogs have focused on the role of the size of the tongue and tongue fat volume in obese patients and the relationship of these measurements to upper respiratory tract (URT) obstructive disorders.^10^, ^11^, ^12^, ^13^ These studies found that obese apnoeic human patients had larger tongues with a higher tongue fat percentage than obese patients without OSA.^10^, ^11^ Severe OSA is also most notably correlated with high tongue fat compared with the other soft tissue structures of the head in humans.^14^ In further investigations, a study of 67 obese OSA patients, who underwent MRI before and after a weight loss programme, found weight loss reduced tongue fat volume and improved their apnoea‐hypopnea index.^15^ In dogs, BOAS is linked to macroglossia as a result of the soft palate becoming displaced dorsally by a larger tongue size, obstructing the pharynx.^12^

In the United Kingdom, owners are becoming less able to identify obese horses.^16^ In 2018 the prevalence of equine obesity was estimated to be 30%–50% with a suspected higher rate in at risk groups such as native breeds, cobs, nonridden and pleasure horses.^5^ There are no studies in the literature exploring the effect of body condition score (BCS) on the structures of the oropharynx, nasopharynx and upper respiratory tract of the horse. Given the increasing prevalence and concerns surrounding equine obesity and the effect of BCS on the tongue in other species, this study aims to investigate if tongue size, soft palate angle or hyoid bone depth in horses is associated with BCS. In addition, it aims to identify the effect of tongue size on the angle of the soft palate. We hypothesised that: (i) increasing BCS will lead to an increase in all measures of tongue size; (ii) increasing BCS will increase basihyoid bone depth; (iii) that increased tongue size will increase soft palate angle.

## 2 MATERIALS AND METHODS

Medical records of horses undergoing computed tomographic (CT) examination of the head at the Leahurst Equine Hospital, University of Liverpool from 25 February 2022 to 8 November 2022 were reviewed. Data on signalment, reason for presentation to the hospital and CT image findings were collated using veterinary practice management software (Tristan, Version 1.8.3.1110). A convenience sample of horses was included if they had undergone a standing CT examination of the head and had BCS recorded in their medical records on the day of the CT examination. Horses that had pathology affecting the caudal mandible, hard palate, soft palate, tongue, or hyoid apparatus were excluded. Horses were also excluded if CT images were distorted by movement.

BCS was performed by one experienced assessor (AMT) on the day of the CT examination, as part of the normal clinical examination, using the scale adhered to by equine charities in the United Kingdom and laid out in the DEFRA code of conduct.^17^ If horses fell equally between the descriptors for each category, half scores were allowed. CT examinations of horses were performed with the horses standing and restrained with intravenous sedation. A combination of acepromazine (AceSedate, Jurox UK Limited) 0.03 mg/kg, morphine sulfate (Martindale Pharma) 0.1 mg/kg and romifidine (Sedivet, Labiana Life Sciences S.A.) 0.06–0.08 mg/kg was used to induce a standardised marked sedation plane in all patients. The head and neck of each patient was supported on a custom‐made, height adjustable, radiolucent carbon fibre table (Bibby Precision Engineering), with the head and neck angle at maximal extension and the horizontal rami of the mandible resting flat on the carbon fibre table. Images were obtained using a 16‐slice, 90 cm bore, multidetector CT scanner (Canon Aquilion, Canon Medical Systems Ltd.). Images were acquired using 16‐row × 1.0 mm detector width, 120 KVp and 300 mAs, 500 mm field of view, 1.0 mm slice thickness, tube rotation time 0.75 s and gantry pitch 0.688. Images were stored on the picture archiving and communications server (Carestream Health Inc) of the University of Liverpool as part of the horse's clinical medical record.

**FIGURE 1:**
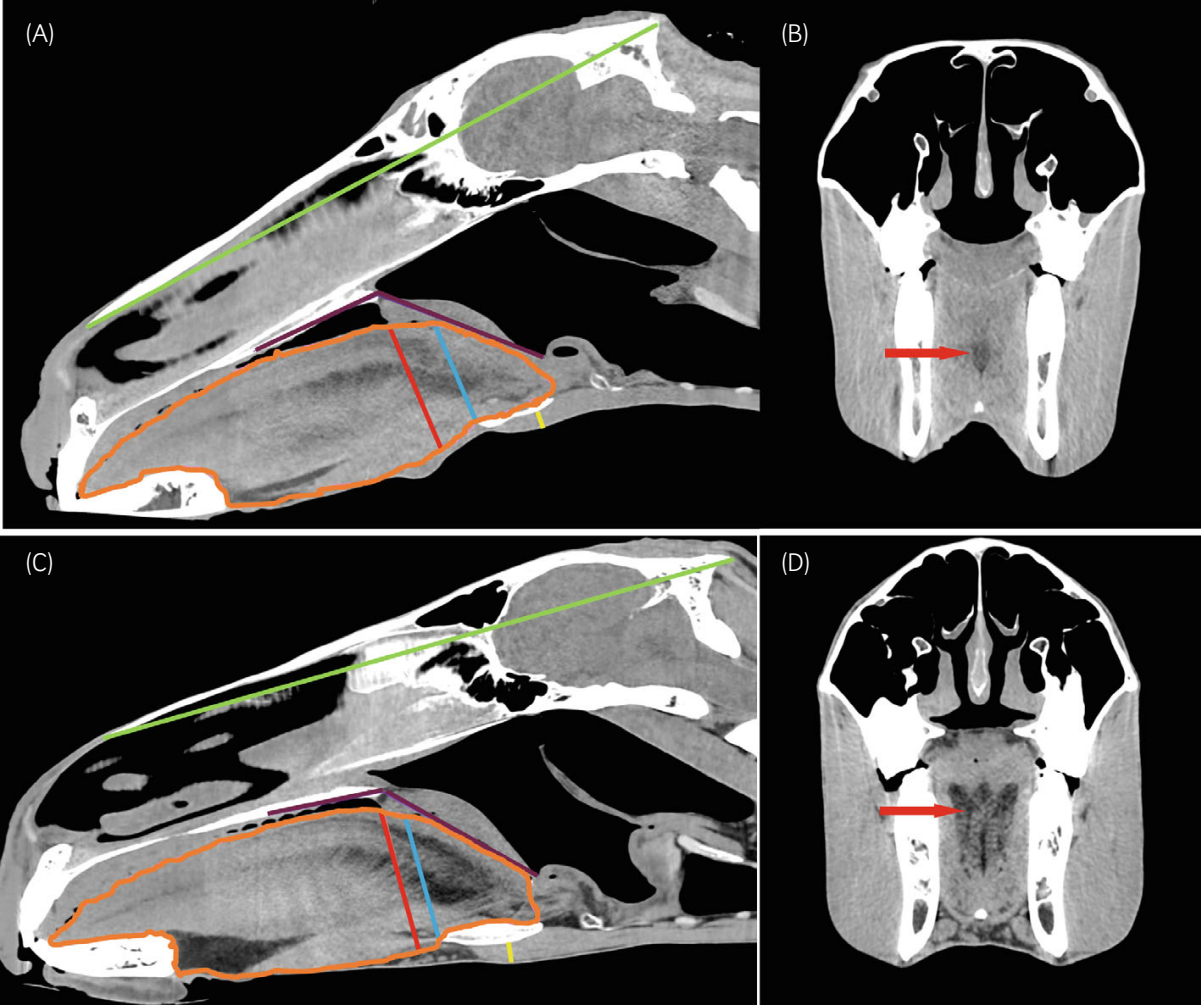
(A–D) 3D multiplanar reconstructions sagittal (A and C) and transverse plane (B and D) CT images in a soft tissue window, WL 35, WW 350 of a horse BCS 1 (A and B) and of a horse BCS 5 (C and D). B and D are at the level of the rostral tip of the lingual process of the basihyoid bone. Sagittal images rostral is on the left and the top is dorsal. Transverse plane images, left is on the right and top is dorsal. Examples of measurements obtained in the sagittal plane images. Head length = green line, soft palate angle with hard palate = purple lines, area of tongue = orange line, DVH of tongue at caudal end of hard palate = red line, DVH of tongue at tip of lingual process = blue line, depth of the basihyoid bone from the ventral skin margin = yellow line. Areas of hypoattenuating fat were clearly seen within the tongue (red arrows). The increased area of fat within the tongue was best appreciated on the transverse plane.

The CT images were analysed by a veterinary undergraduate student in their 3rd year of study (HSB), supervised by a Diplomate of the American College of Veterinary Sports Medicine and Rehabilitation experienced in CT image interpretation (AMT). A training period of several weeks was given and the repeatability of a sample of measurements was tested prior to the study measurements being obtained. Images were viewed on a computer monitor, using DICOM software (HOROS™, GNU Lesser General Public License, Version 3.0, LGPL 3.0) in multiplanar reconstructions. The sagittal midline view was obtained by aligning the sagittal plane to the vomer bone. Midline sagittal reconstructions were used to obtain measurements using calibrated measuring tools within the viewing software. Six measurements were obtained for the study (Figure 1). Each measurement was recorded twice by the same observer (HSB), with a 1‐week time interval between repeat measurements, and a mean value calculated. The measurements obtained were as follows:

1. A region of interest is drawn around the tongue margins to acquire a sagittal midline tongue area. The ventral tongue margin was the ventral aspect of the geniohyoideus muscle. The caudal margin was drawn around the tongue muscles at the level of the caudal part of the basihyoid bone. Ventral and rostral aspects encompassed the intrinsic and extrinsic tongue muscles and the midline ventral fat pad.
2. The dorsoventral height (DVH) of the tongue was measured in two places:
  - The perpendicular distance from the tip of the lingual process to the dorsal border of the tongue.
  - The perpendicular distance from the ventral aspect of the tongue to the dorsal border of the tongue at the level of the caudal edge of the hard palate.
3. The angle of the soft palate with the hard palate (SPA) was measured from the ventral aspect of the hard palate to the soft palate. The points were placed on the long axis of the hard palate, the most caudal point of the hard palate and the free end of the soft palate where it meets the epiglottis.
4. The distance from the caudal edge of the lingual process of the basihyoid bone, immediately rostral to the bifurcation, to the ventral skin margin.
5. The distance from the most rostral aspect of the nasal bone to the occipital protuberance as a reference for skull length.

Following analysis of the initial results, measurement of head length, defined as the length from the rostral part of the nasal bone to the external occipital protuberance, was made in a subset of horses in which the occipital protuberance was visible. Some scans required minor adjustments from the midline to ensure the longest portion of the basihyoid bone was visible.

Following further consideration of the results and taking into account the previously reported effect of head position on radiographic assessment of laryngeal position,^18^ an additional measure of head angle was recorded in the 44 horses that met the initial inclusion criteria. The measurement was performed three times by the same observer (AT), 2 days apart for each measure set and a mean value was calculated. The angle of the head was defined as the angle between the intermandibular space and the most rostral portion of the ventral aspect of the neck.

Subjective assessment of the fat distribution within the tongue and pharyngeal region was noted during the study, with fat determined using CT attenuation grey scale and Hounsfield units (HU) (−190, −30 HU).^19^ In addition, in order to confirm fat distribution the tongue and larynx of a single horse of BCS 4, euthanised for reasons relating to colic and whose body was donated for teaching purposes, was examined on gross post‐mortem and histological evaluation. This horse was not part of the CT image analysis group.

Statistical analyses were carried out using a dedicated statistical software package (Minitab, Version 19.2020.1 and IBM SPSS). The Ryan–Joiner normality test was employed to evaluate whether each variable followed a normal distribution. To identify whether head length or age was associated with the CT measurements the Spearman's rank correlation coefficient was calculated. The Mann–Whitney *U* test was used to assess whether sex was associated with any CT measurements. When comparing with BCS, if the dependent variable was normally distributed the one‐way analysis of variance (ANOVA) test was used, if there was a non‐normal distribution a Kruskal–Wallis *H* test was chosen. To test for associations between tongue measurements with soft palate angle and basihyoid bone depth the Spearman's rank correlation coefficient was calculated. Spearman's rank correlation coefficient was calculated to test for associations between head angle and soft palate angle and basihyoid bone depth. Calculation of the intraclass correlation coefficients (ICC) and their 95% confidence intervals (95% CI) was used to compare first and second measurements to assess the repeatability of methods, using a single‐measurement, absolute‐agreement, two‐way mixed‐effects model. *p* ≤ 0.05 was considered significant for all analyses.

## 3 RESULTS

### 3.1 Study population

**FIGURE 2:**
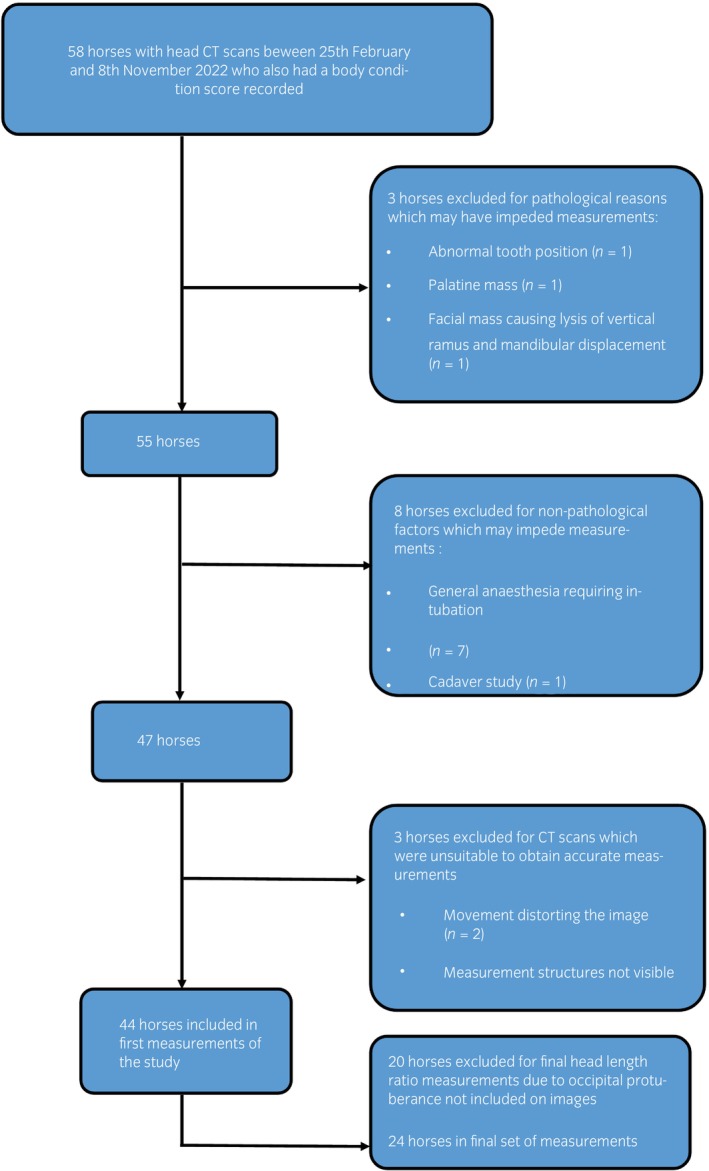
Flow chart illustrating inclusion and exclusion criteria of horses at each stage of the study.

Of the records searched, 44 of 58 horses met the inclusion criteria for the calculation of initial measurements. The addition of head length ratio, in the second set of calculations, resulted in 24/58 horses meeting the final inclusion criteria (Figure 2).

Horses consisted of a variety of breeds, Cob (*n* = 6), Connemara (*n* = 2), Highland pony (n = 2), Irish draught (*n* = 1), Irish Sports Horse (*n* = 7), Selle Français (*n* = 1), Thoroughbred (*n* = 4), Warmblood (*n* = 8), Welsh pony (*n* = 2), crossbreed (*n* = 6) and unknown breed (*n* = 5). Horses ranged from 2 to 23.5 years (median 11.5 years; IQR 6–14.8 years); 26/44 (59.09%) were geldings, 17/44 (38.64%) were mares and one (2.27%) was a stallion. BCS ranged from 1 to 5; BCS 1 (*n* = 1), BCS 2 (*n* = 2), BCS 2.5 (n = 2), BCS 3 (*n* = 17), BCS 3.5 (*n* = 4), BCS 4 (*n* = 16) and BCS 5 (n = 2). (Summary of signalment given in Table S1).

Distribution of variables is provided in Table S2. Repeatability of all measures was found to be excellent (Data S1).

### 3.2 Head length

There was no significant correlation between head length and angle of the soft palate (*p* = 0.619) or basihyoid depth (*p* = 0.654). A significant correlation was identified between head length and tongue area (Spearman's *r* = 0.840; *p* < 0.001); and head length and DVH of the tongue at the level of the hard palate (Spearman's *r* = 0.549; *p* = 0.005) and DVH of the tongue at the level of the lingual process of the basihyoid bone (Spearman's *r* = 0.454; *p* = 0.026) (Table S3). Therefore, increased head length led to an increase in all three tongue measurements.

To reduce the effect of varying head length, all further analysis involving measurements of the tongue were carried out using tongue measures as proportions of head length in horses in which this measurement was available (24/44). These proportions were calculated by dividing the tongue measurement by head length. Tongue area as a proportion of head length was a normally distributed variable (*p* > 0.1) whereas DVH of the tongue at the level of the hard palate as a proportion of head length was non‐normally distributed (*p* = 0.03).

Head length was also significantly correlated with head angle (Spearman's *r* = 0.419; *p* = 0.04).

### 3.3 Body condition score

Summary statistics were calculated for all measured variables based on BCS groups (Tables S2 and S3). There was no significant difference in tongue area as a proportion of head length (*p* = 0.197) or DVH of the tongue at the level of the hard palate as a proportion of head length (*p* = 0.086). Additionally, there was no significant difference in mean DVH of the tongue at the level of the lingual process of the basihyoid bone as proportion of head length between BCS group (*p* = 0.3).

Increasing BCS did; however, lead to a significant increase in soft palate angle (*p* = 0.02, *F* 2.93). In addition, increasing BCS led to a significant increase in median basihyoid depth (*p* = 0.006, *H* 17.92).

**FIGURE 3:**
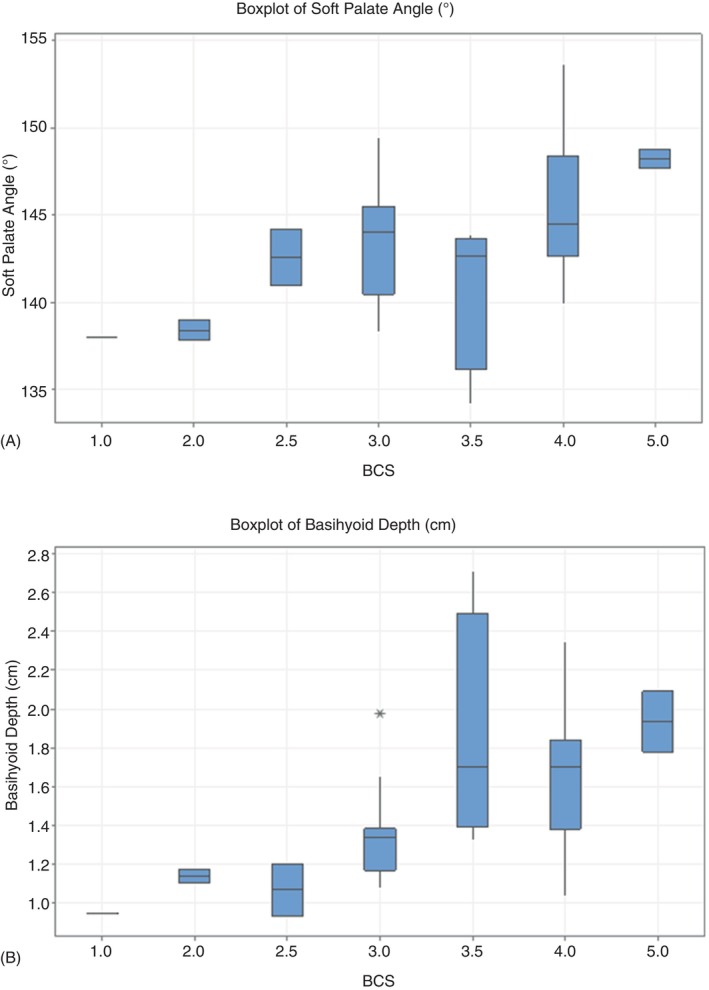
(A). Box and whisker plot showing the distribution of soft palate angle measurements within each body condition score. BCS, body condition score. (B). Box and whisker plot showing the distribution of basihyoid depth measurements (cm) within each body condition score. BCS, body condition score; cm, centimetres. * = outlier.

Box and whisker plots illustrate the distribution of each variable in relation to body condition scores. Statistically significant variables in relation to BCS are shown in Figure 3. (Graphs for nonsignificant variables are given in Figure S1).

### 3.4 Tongue size

**FIGURE 4:**
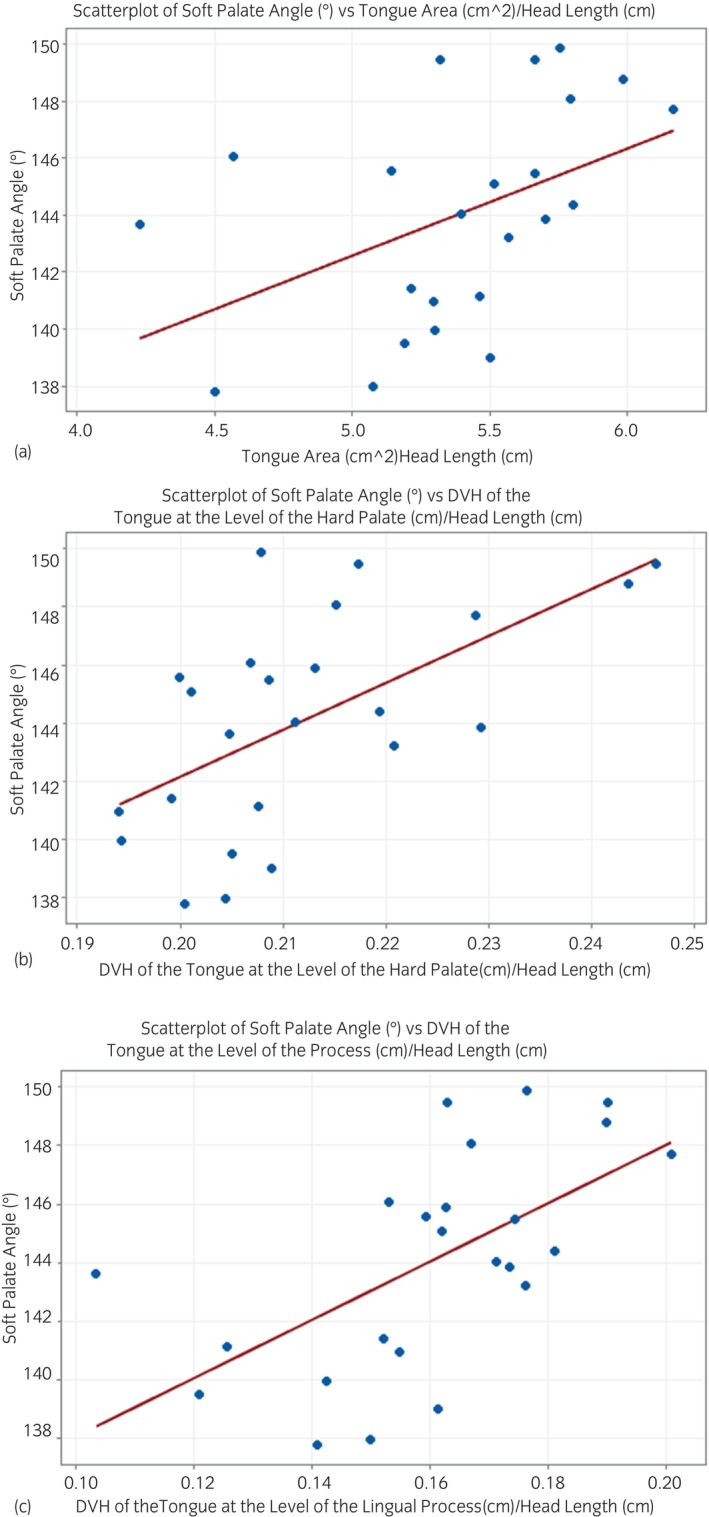
(A) A scatterplot showing the relationship between soft palate angle (°) and tongue area (cm^2^) as a proportion of head length (cm). cm, centimetres. (B) A scatterplot showing the relationship between soft palate angle (°) and dorsoventral height of the tongue at the level of the hard palate (cm) as a proportion of head length (cm). DVH, dorsoventral height; cm, centimetres. (C) A scatterplot showing the relationship between soft palate angle (°) and dorsoventral height of the tongue at the level of the lingual process of the basihyoid bone (cm) as a proportion of head length (cm). DVH, dorsoventral height; cm, centimetres.

A significant moderate correlation between soft palate angle and tongue area as a proportion of head length (Spearman's *r* = 0.544; *p* = 0.007) was identified. Additionally, a significant moderate correlation was identified between soft palate angle and DVH of the tongue at the level of the hard palate as a proportion of head length (Spearman's *r* = 0.562; *p* = 0.004) and with DVH of the tongue at the lingual process of the basihyoid bone as proportion of head length (Spearman's *r* = 0.690; *p* < 0.001) (Figure 4; Table S4).

There was a significant moderate positive correlation between basihyoid depth and DVH of the tongue at the level of the hard palate as a proportion of head length (Spearman's *r* = 0.491; *p* = 0.015). In addition, there was significant moderate positive correlation between basihyoid depth and the DVH of the tongue at the level of the lingual process of the basihyoid bone as a proportion of head length (Spearman's *r* = 0.415; *p* = 0.044). The correlation between basihyoid depth and tongue area as a proportion of head length was of borderline significance (*p* = 0.05; r = 0.411) (Table S5).

### 3.5 Head angle

There was no significant correlation between head angle and basihyoid bone depth (Spearman's *r* = 0.139; *p* = 0.4), soft palate angle (Spearman's *r* = 0.045; *p* = 0.8) or any measures of the tongue as a proportion of head length.

### 3.6 Sex

The median value for all variables was lower in females than males except for basihyoid depth which was higher in females. There was no significant difference for any of the measured variables between male and female horses (Tables S6 and S7).

### 3.7 Age

A significant correlation was found between head angle and age (Spearman's *r* = −0.324; *p* = 0.03). No significant correlation was found between age and any other measured variable (Table S8).

### 3.8 Subjective visual assessment of CT images and post‐mortem specimen observations

**FIGURE 5:**
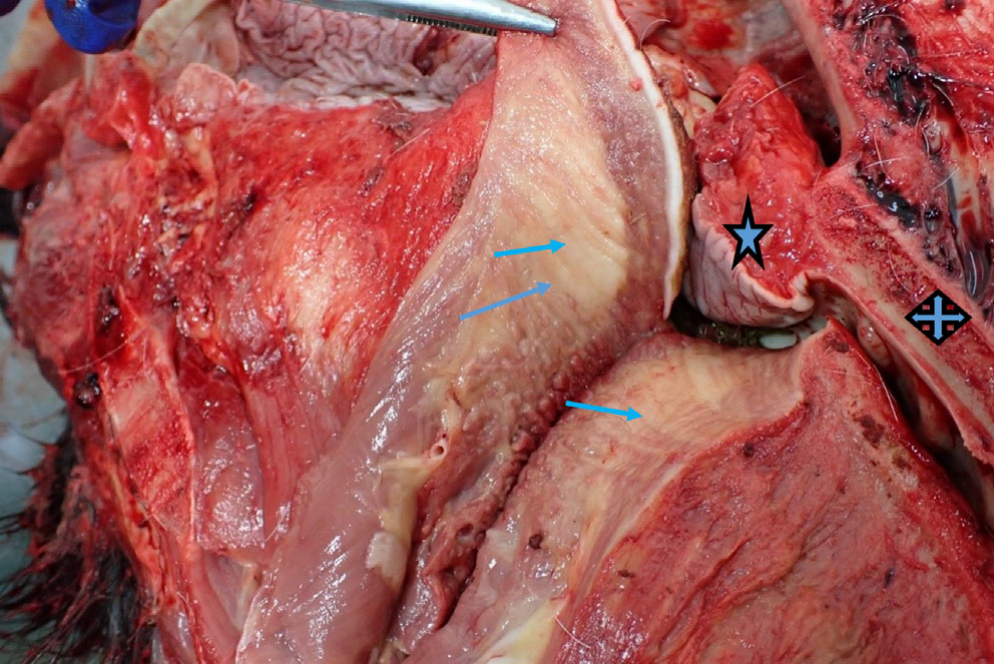
Photograph of gross post‐mortem dissection of the cut section of the tongue of an obese horse unrelated to the CT study. Adipose tissue is identified infiltrating the intrinsic muscles of the tongue (arrows). Blue star = Soft palate; blue cross = hard palate.

Multiple regions of fat deposits were identified in the laryngeal and pharyngeal regions on CT images: There was a large hypoattenuating area immediately caudal to the mandibular symphysis and ventral to the rostral part of the tongue, visible on the sagittal midline CT images that correlated to fat on gross post‐mortem examination. There was an additional notable amount of fat diffusely distributed throughout the intrinsic muscles in the posterior portion of the tongue, best appreciated on transverse plane images. The soft palate appeared to vary in shape and thickness between horses although it did not contain tissue of fat attenuation. There was a large variation in the volume of fat in the sub‐epiglottic space; however, this study did not investigate the cause or trend of these changes. Subcutaneous fat could also be clearly seen in the horses with a higher body condition score ventral to the basihyoid bone and ventral to the thyroid cartilages and trachea. The post‐mortem dissection and histology confirmed abundance of fat laced between the fibres of the intrinsic muscles of the tongue and surrounding the extrinsic muscles the genioglossus and the geniohyoideus (Figure 5).

## 4 DISCUSSION

Despite the well‐studied effects of obesity on the URT in humans and dogs, to the authors' knowledge, this is the first study that objectively measures the effect of body condition score on structures of the URT and the tongue in the horse. In addition, veterinary anatomy text books have very limited written reference to the abundance of fat within the equine tongue and surrounding the larynx despite this being clearly visible on gross post‐mortem examination.^20^, ^21^ Fat is also clearly visible interlaced within the intrinsic muscles of the tongue on CT images although reference texts books on the latter topic fail to specifically label the adipose tissue present.^22^ Perhaps the lack of emphasis of fat in and around these structures is due to growing levels of obesity in equids seen in years subsequent to the publication of some of the standard veterinary anatomy texts or simply a dismissal of the potential importance of this tissue within the tongue with regards to biomechanics.

The body condition scoring system used in this study was adapted slightly from the referenced scoring system^17^ to reflect horses that fell equally between two categories. This is the system used in the Horse Trust Weigh to Win BCS scheme run in the United Kingdom since 2019 that assesses show horses for BCS whilst in the show ring.^23^ This numbering scale is used because it is anecdotally perceived that lay equestrians find a scale of 0–5 with half scores easier to understand than the 1–9 scale commonly used in the veterinary literature.^24^ The author undertaking the BCS has undergone standardised training in the modified scoring system used in this study as part of the Weigh to Win judging criteria alongside numerous other UK‐based veterinary surgeons who also use this scale.

Interestingly, our first hypothesis was disproved in that we did not identify a significant relationship between the BCS and measures of the tongue. Given that there is a strong correlation with body mass index/body condition score in other species and measures of the tongue, this result was surprising and perhaps is a spurious result due to the small sample size of the horses included in the tongue measures once head length had been corrected for. Despite the lack of correlation between BCS and measures of the tongue, it is noted that an increased DVH of the tongue at two locations, expressed as a proportion of head length, was associated with an increase in basihyoid depth. This is a slightly curious finding in that if measures of the tongue alone were impacting basihyoid depth, then it may be reasonable to assume that an increase in tongue height would result in a more ventrally located basihyoid depth due to localised mass effect which was not the identified in this study. Despite the height of the tongue not being statistically related to the BCS there was a general direction of increasing tongue measurements with BCS when plotted on a scatter graph. Two possible explanations for the lack of correlation of BCS with tongue measurements in this study were considered. In humans a link between visceral fat in the abdomen and tongue fat has been reported.^10^ Perhaps visceral fat in horses may be better correlated with tongue measurements than subcutaneous fat measures and external body condition score would correlate better with subcutaneous fat. Additional work to include correlation of tongue fat with retroperitoneal or subcutaneous fat would be required to evaluate this possibility. Second, the low number of horses included in the tongue measurements, after correction for head length, may have influenced the results due to a lower study power. Therefore, the influence of BCS on tongue measures and specifically fat volumes in the tongue requires further work.

Our second hypothesis can be accepted in that the depth of the basihyoid bone was strongly correlated with the BCS. This is potentially a very important finding because a more ventral basihyoid bone location, as measured on ultrasound examination, has been linked to the occurrence of intermittent dorsal displacement of the soft palate (iDDSP) at exercise in Thoroughbred horses.^25^ In addition, the laryngeal tie forward procedure used to treat iDDSP results in a more dorsal position of the base of the basihyoid bone as measured on radiographs.^26^ The application of a tongue tie in standing horses at rest has also been shown to result in a significant increase in depth of the basihyoid bone from the ventral skin when measured ultrasonographically^27^ and tongue ties have been shown to improve the racing performance in certain populations of horses.^28^ The exact aetiopathogenesis of iDDSP is unknown with multifactorial components likely.^29^ Young horses that continue to mature may see an improvement or complete resolution in the condition as they age.^30^ Considering our observed effect of BCS on basihyoid bone depth, it is postulated that perhaps adipose tissue acts as a natural supporter of the basihyoid bone and a lack of fat in immature or elite fit horses removes this protection in horses exercising at speed. As far as we were aware none of the horses in this study had any history of iDDSP and therefore further work is required to understand the significance of body condition score and fat volume in the tongue, surrounding the hyoid apparatus and in the nasopharynx in horses that suffer from this condition.

The results also upheld our third hypothesis in that the SPA was related to measures of the tongue once adjusted for head length. Surprisingly the SPA was also significantly related to BCS even though actual tongue size was not. This may have been due to increased fat depth ventral to the thyroid cartilages and epiglottis, or it could still be related to the fat in the tongue which is the most likely explanation given the relationship of SPA to measures of the tongue. Further work is now required to establish the significance of this finding in the clinical setting. It is speculated that the angle of the soft palate may be important when considering oral palatal seal and palatal stability in exercising horses. Perhaps there is an optimum tongue size and SPA that exists in the horse in relation to the oral palatal seal.

In this study, there was no correlation of head angle with either soft palate angle or basihyoid bone depth. This is most likely due to the standardised positioning for CT examination, with the horses in maximal head–neck extension. The radiographic position of the larynx has been shown to be affected by head positions of neutral, extended and flexed as reported by McCluskie et al.^18^ All the horses in the current study fell within the category of extended (>115 degrees). It would be interesting to repeat the current study with the head in a flexed and neutral position; however, this is not practically possible when using standing CT in horses.

Surprisingly, head angle was significantly correlated with head length and with age of horse. It was subjectively easier to position a horse with a longer head and neck length in an extended position on the CT table than a horse with a shorter head and neck and so this may explain the apparent association with head length observed. Younger horses had an overall greater head angle when positioned in full extension than older horses. This is a curious finding, and it is postulated that this may be related to the elasticity of the ligaments at the occipital atlantoaxial junction. A previous study has shown that mineralisation of the longitudinal odontoid ligaments, as viewed on CT, is significantly associated with age.^31^ This may have resulted in an overall subtle reduction in range of motion of the upper cervical region in horses in our study which was of sufficient magnitude to correlate with age although too small a change to affect laryngeal position.

### 4.1 Limitations

This study contains several limitations. The study did not investigate whether breed was a confounding factor due to insufficient cases within each breed. The sample size was small and was further reduced where adjustments were made for the effect of head length on tongue measures. The investigator collecting CT measurements was not blinded to the BCS which may have introduced a level of unconscious bias. Further limitations include the fact that the measurements were only made on a single sagittal plane. All the CT scans were obtained with horses sedated. Sedation may also influence volume of the nasopharynx in horses and it is acknowledged that this may have affected the overall position of structures evaluated; however, all horses were evaluated under a similar deep plane of sedation using the same sedation protocol, so it would not be possible to determine if sedation had affected individual results differently in this study.

## 5 CONCLUSIONS

The current study shows that the angle of the soft palate in relation to the hard palate, in a sedated horse with the head in maximal extension, is related to BCS. A more dorsal resting position of the basihyoid bone in relation to the ventral skin surface is also seen in relation to increased BCS. Additionally, our results found an increase in the angle of the soft palate occurred with an increase in tongue height/head length ratio. Further investigations into the effect of BCS on the soft tissue structures of the upper airway and the implications of these changes with respect to dynamic URT conditions of the horse are warranted.

## FUNDING INFORMATION

None.

## CONFLICT OF INTEREST STATEMENT

The authors declare no conflicts of interest.

## AUTHOR CONTRIBUTIONS

**Alison M. Talbot:** Conceptualization; methodology; software; data curation; investigation; validation; supervision; resources; project administration; visualization; writing – original draft; writing – review and editing; formal analysis. **Hannah Shanks‐Boon:** Methodology; data curation; software; investigation; validation; formal analysis; writing – original draft; writing – review and editing. **Christopher M. Baldwin:** Conceptualization; investigation; validation; supervision; methodology; visualization; project administration; writing – review and editing; resources. **Harriet Barnes:** Conceptualization; methodology; supervision; project administration; validation; visualization; writing – review and editing; investigation; resources. **Thomas W. Maddox:** Conceptualization; methodology; project administration; supervision; visualization; writing – review and editing; investigation; validation; resources; formal analysis.

## DATA INTEGRITY STATEMENT

Alison Talbot had full access to all the data in the study and takes responsibility for the integrity of the data and the accuracy of data analysis.

## ETHICAL ANIMAL RESEARCH

University of Liverpool Ethics Review Committee, approval number VREC1226.

## INFORMED CONSENT

Owners gave informed consent for generic retrospective analysis of clinical data for research purposes on consent forms on admission.

### PEER REVIEW

The peer review history for this article is available at https://www.webofscience.com/api/gateway/wos/peer-review/10.1111/evj.14445.

## Supporting information


**Data S1.** Repeat measurements.

**Figure S1.** Box and whisker plots showing the distribution of measured variables (head length, tongue area, dorsoventral height of the tongue at the level of the hard palate as a proportion of head length and dorsoventral height of the tongue at the level of the lingual process of the basihyoid bone as a proportion of head length) that were not statistically significant when correlated to body condition score. DVH, dorsoventral height; BCS, body condition score; cm, centimetres.

**Table S1.** A table showing body condition score and signalment of each patient including age, breed and sex. BCS, body condition score; y, years; m, months; X, crossbreed.

**Table S2.** Distribution of variable—Results of Ryan–Joiner normality test for all measured variables.

**Table S3.** Summary of results from Spearman's rank correlation comparing head length with tongue measurements, soft palate angle, basihyoid depth and head angle.

**Table S4.** Results of Spearman's rank correlation test between tongue measurements and soft palate angle.

**Table S5.** Results of Spearman's rank correlation between tongue measures and basihyoid depth.

**Table S6.** Summary of results from Mann–Whitney *U* test comparing all measured variables between groups of male and female.

**Table S7.** Male and female summary statistics for all measured variables. DVH, dorsoventral height; cm, centimetres.

**Table S8.** Results of Spearman's rank correlation between age and all computed tomography measured variables.

## Data Availability

The data that support the findings of this study are available from the corresponding author upon reasonable request. Open sharing exemption granted by the editor for this retrospective clinical report.
