# Supplementary material for: Soft palate angle and basihyoid depth increase with tongue size and with body condition score in horses
Source: Equine Vet J. 2025 Jan 2;57(4):967–76. doi: 10.1111/evj.14445 (PMC12135754; doi:10.1111/evj.14445)
Supplement: Supplementary file 1 — Data S1. Repeat measurements. [file EVJ-57-967-s007.pdf]

Equine Veterinary Journal BEVA

Data S1

Repeat Measurements

Repeatability of all measures was found to be excellent, with intraclass correlation coefficients of head length = 0.94 (95%CI 0.86-0.93; P<0.01); SPA=0.952 (95%CI 0.91-0.97; P< 0.001); tongue area= 0.99 (95%CI 0.99-0.99; P<0.01); DVH of the tongue hard palate=0.925 (95%CI 0.86-0.95; P<0.01); DVH of the tongue lingual process=0.93 (95% CI 0.91-0.98; P<0.01) and basihyoid depth= 0.979 (95%CI 0.96-0.98; P<0.01). Head angle 0.996 (95% CI 0.994-0.998; P<0.01).
