# Supplementary material for: Soft palate angle and basihyoid depth increase with tongue size and with body condition score in horses
Source: Equine Vet J. 2025 Jan 2;57(4):967–76. doi: 10.1111/evj.14445 (PMC12135754; doi:10.1111/evj.14445)
Supplement: Supplementary file 2 — Figure S1. Box and whisker plots showing the distribution of measured variables (head length, tongue area, dorsoventral height of the tongue at the level of the hard palate as a proportion of head length and dorsoventral height of the tongue at the level of the lingual process of the basihyoid bone as a proportion of head length) that were not statistically significant when correlated to body condition score. DVH, dorsoventral height; BCS, body condition score; cm, centimetres. [file EVJ-57-967-s010.pdf]

Equine Veterinary Journal BEVA

Figure S1. Box and whisker plots showing the distribution of measured variables (head length, tongue area, dorsoventral height of the tongue at the level of the hard palate as a proportion of head length and dorsoventral height of the tongue at the level of the lingual process of the basihyoid bone as a proportion of head length) that were not statistically significant when correlated to body condition score. DVH- dorsoventral height; BCS- body condition score; cm- centimetres.

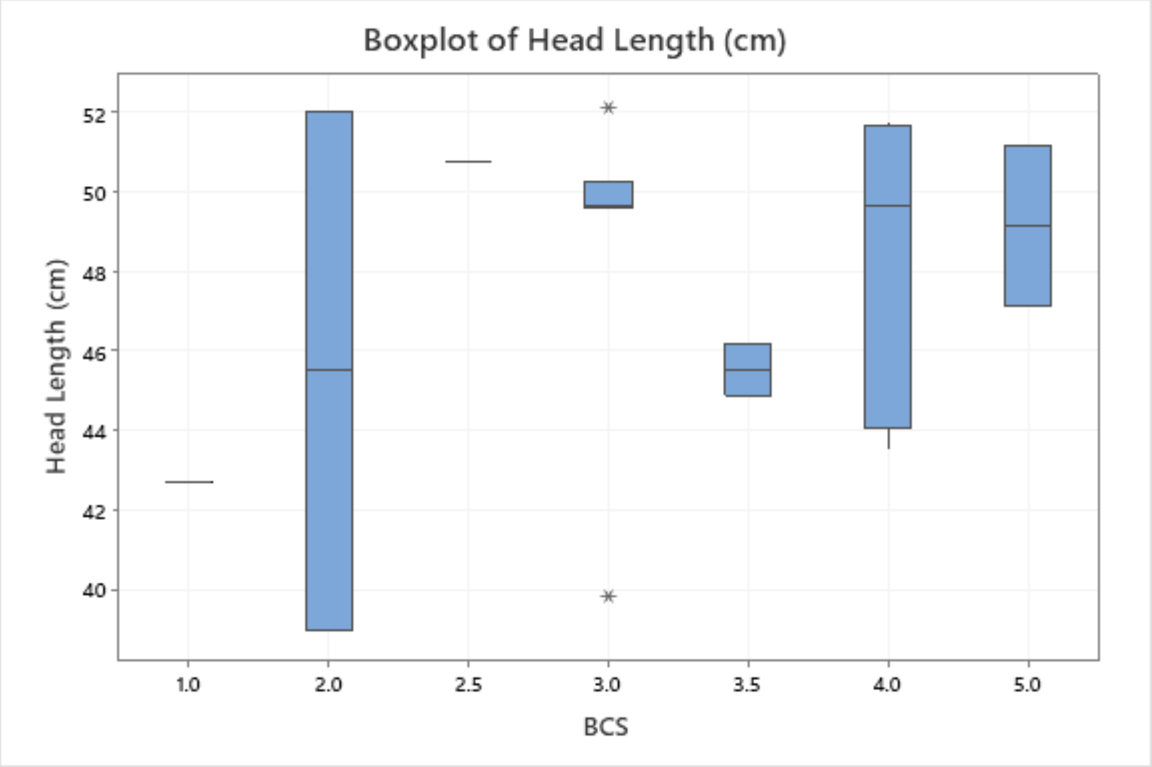

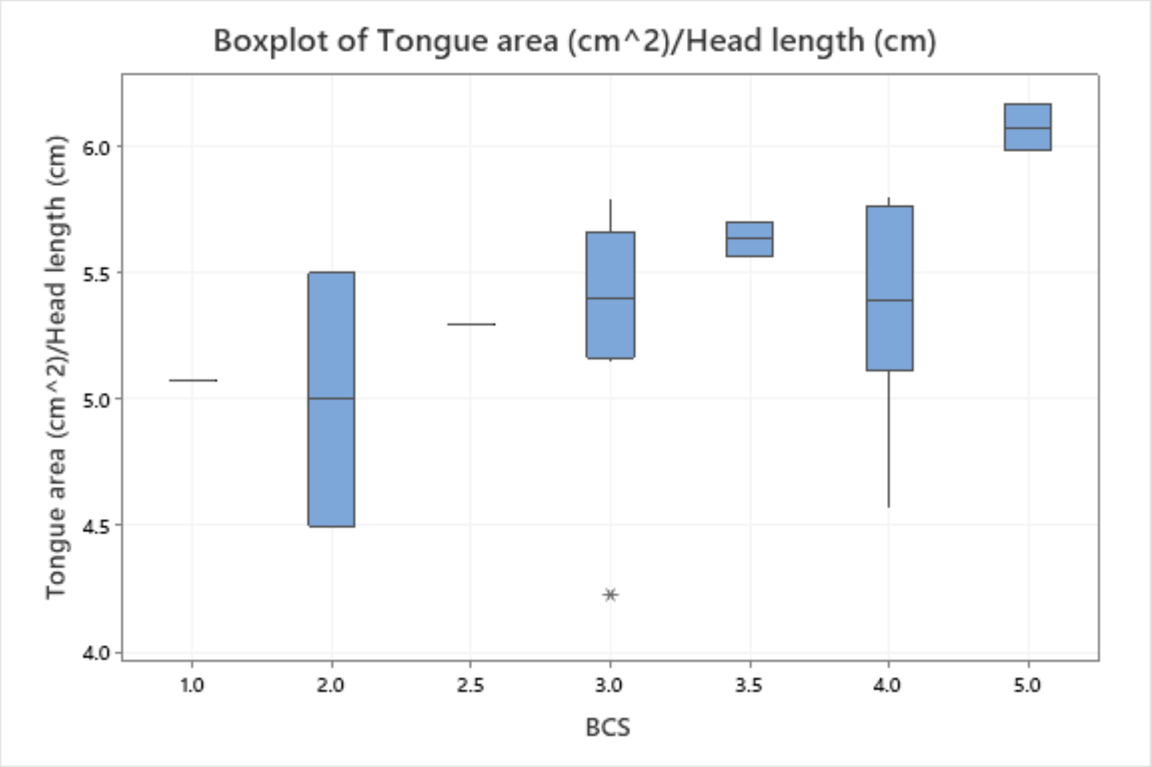

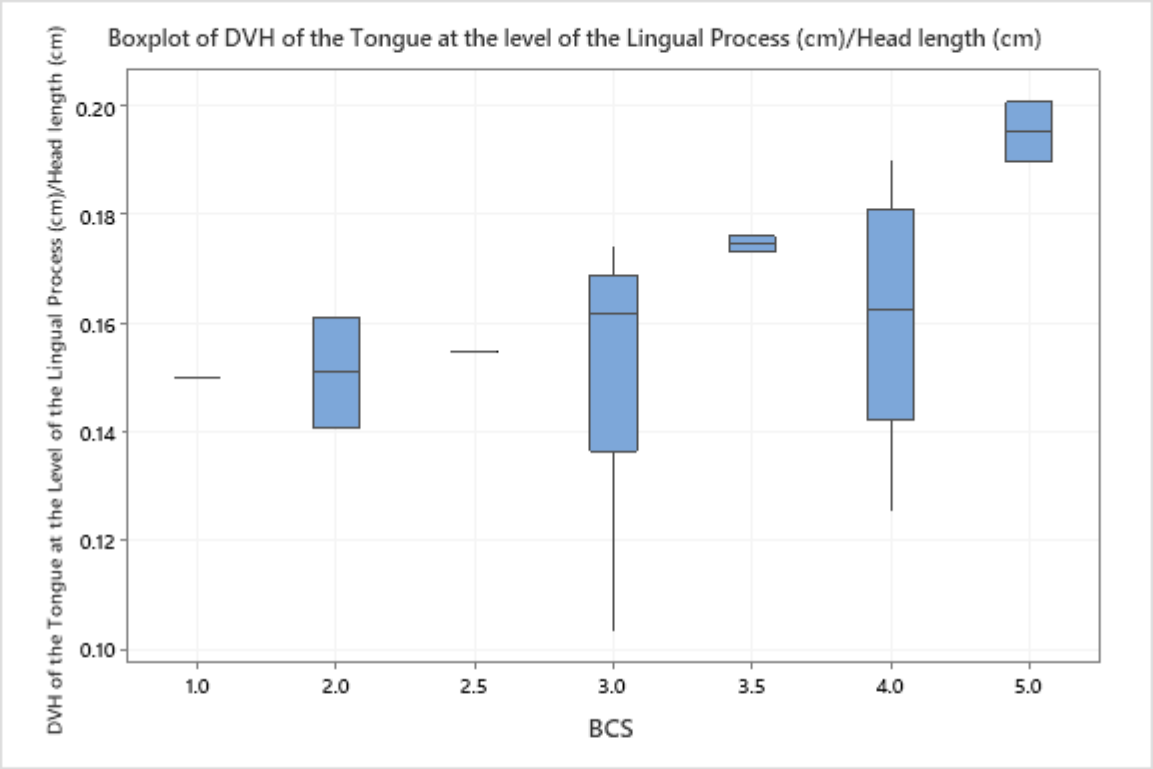
Boxplot of DVH of the Tongue at the level of the Lingual Process (cm)/Head length (cm)
DVH of the Tongue at the Level of the Lingual Process (cm)/Head length (cm)
0.20
0.18
0.16
0.14
0.12
0.10
1.0
2.0
2.5
3.0
3.5
4.0
5.0
BCS
