# Supplementary material for: Soft palate angle and basihyoid depth increase with tongue size and with body condition score in horses
Source: Equine Vet J. 2025 Jan 2;57(4):967–76. doi: 10.1111/evj.14445 (PMC12135754; doi:10.1111/evj.14445)
Supplement: Supplementary file 3 — Table S1. A table showing body condition score and signalment of each patient including age, breed and sex. BCS, body condition score; y, years; m, months; X, crossbreed. [file EVJ-57-967-s001.pdf]

Equine Veterinary Journal BEVA

Table S1: A table showing body condition score and signalment of each patient including: age, breed and sex. BCS- body condition score; y- years; m- months; X- crossbreed.

Horse number	BCS (/5)	Breed	Age	Sex
1	3.5	Unknown	16y	Mare
2	3	Warmblood	4y	Gelding
3	4	Cob	14y 1m	Gelding
4	3	Thoroughbred	18y 8m	Mare
5	3	Dutch Warmblood	5y	Gelding
6	4	Warmblood	10y 5m	Stallion
7	3.5	Unknown	18y	Mare
8	3.5	Connemara	18y	Mare
9	3.5	Cob	10y	Gelding
10	1	Unknown	22y 9m	Mare
11	4	Irish Sports Horse	15y	Gelding
12	5	Cob	20y	Gelding
13	4	Irish Sports Horse	13y	Mare
14	4	Connemara	10y	Mare
15	4	Welsh X	23y	Gelding
16	4	Warmblood X	13y	Mare
17	3	Sports Horse	7y 3m	Gelding
18	2	Thoroughbred	3y 5m	Mare
19	4	Warmblood	9y	Gelding
20	4	Irish Draught X	13y	Gelding
21	4	Unknown	14y 7m	Gelding
22	3	Dutch Warmblood	14y 3m	Mare
23	4	Warmblood	3y	Gelding
24	3	Irish Sports Horse	12y	Gelding
25	2	Thoroughbred	6y	Gelding
26	3	Irish Sports Horse	14y 3m	Mare
27	3	Thoroughbred	15y	Gelding
28	3	Irish Draught X Thoroughbred	14y 1m	Gelding
29	4	Irish cob	15y 7m	Gelding

30	3	Connemara X	6y	Gelding
31	3	Warmblood	9y	Mare
32	3	Irish Sports Horse	3y	Gelding
33	3	Irish Draught X Thoroughbred	23y 6m	Mare
34	3	Irish Draught	12y	Gelding
35	3	Cob	2y	Gelding
36	5	Highland	11y	Gelding
37	4	Welsh C	5y	Gelding
38	4	Welsh A	15y	Gelding
39	3	Pony	10y 4m	Mare
40	2.5	Warmblood	6y	Mare
41	3	Selle Francais	10y	Mare
42	4	Cob	10y	Mare
43	4	Highland	12y	Gelding
44	2.5	Irish Sports Horse	6y	Gelding
