# Supplementary material for: Soft palate angle and basihyoid depth increase with tongue size and with body condition score in horses
Source: Equine Vet J. 2025 Jan 2;57(4):967–76. doi: 10.1111/evj.14445 (PMC12135754; doi:10.1111/evj.14445)
Supplement: Supplementary file 4 — Table S2. Distribution of variable—Results of Ryan–Joiner normality test for all measured variables. [file EVJ-57-967-s003.pdf]

Equine Veterinary Journal BEVA

Table S2. Distribution of variable - Results of Ryan-Joiner normality test for all measured variables.

**Variable**	**Number of values**	**Mean**	**Standard deviation**	**Ryan-Joiner correlation coefficient**	***P*-value**	**Distribution**
BCS	44	3.386	0.762	0.992	>0.100	Normal
Head length (cm)	24	48.020	3.830	0.932	**<0.010**	Non-normal
Soft palate angle (˚)	44	143.700	3.909	0.992	>0.100	Normal
Tongue area (cm^2)	41	260.500	34.160	0.955	**<0.010**	Non-normal
Tongue area (cm^2)/head length (cm)	23	5.382	0.468	0.965	>0.100	Normal
DVH of the tongue at the level of the hard palate (cm)	44	10.290	0.913	0.982	>0.100	Normal
DVH of the tongue at the level of the hard palate (cm)/head length (cm)	24	0.212	0.014	0.949	**0.033**	Non-normal
DVH of the tongue at the level of the lingual process (cm)	44	7.861	1.173	0.971	**0.039**	Non-normal
DVH of the tongue at the level of the lingual process (cm)/head length (cm)	24	0.161	0.023	0.980	>0.100	Normal
Basihyoid depth (cm)	44	1.501	0.401	0.964	**0.014**	Non-normal
Head angle (˚)	44	169.2	5.642	0.982	>0.100	Normal

Statistically significant results highlighted in bold. DVH- dorsoventral height; BCS- body condition score; cm- centimetres.
