# Supplementary material for: Soft palate angle and basihyoid depth increase with tongue size and with body condition score in horses
Source: Equine Vet J. 2025 Jan 2;57(4):967–76. doi: 10.1111/evj.14445 (PMC12135754; doi:10.1111/evj.14445)
Supplement: Supplementary file 5 — Table S3. Summary of results from Spearman's rank correlation comparing head length with tongue measurements, soft palate angle, basihyoid depth and head angle. [file EVJ-57-967-s005.pdf]

Equine Veterinary Journal BEVA

Table S3. Summary of results from Spearman's rank correlation comparing head length with tongue measurements, soft palate angle, basihyoid depth and head angle.

**Variable**	**Number of values**	**Spearman's r**	***P*-value**
Soft palate angle (˚)	24	0.107	0.619
Tongue area (cm^2)	23	0.840	**<0.001**
DVH of the tongue at the level of the hard palate (cm)	24	0.549	**0.005**
DVH of the tongue at the level of the lingual process (cm)	24	0.454	**0.026**
Basihyoid depth (cm)	24	-0.097	0.654
Head Angle (^{0})	24	0.419	**0.042**

Statistically significant results highlighted in bold. DVH- dorsoventral height; cm-centimetres.
