# Supplementary material for: Soft palate angle and basihyoid depth increase with tongue size and with body condition score in horses
Source: Equine Vet J. 2025 Jan 2;57(4):967–76. doi: 10.1111/evj.14445 (PMC12135754; doi:10.1111/evj.14445)
Supplement: Supplementary file 6 — Table S4. Results of Spearman's rank correlation test between tongue measurements and soft palate angle. [file EVJ-57-967-s006.pdf]

Equine Veterinary Journal BEVA

Table S4. Results of Spearman's rank correlation test between tongue measurements and soft palate angle.

**Variable**	**Number of values**	**Spearman's r**	***P*-value**
Tongue area (cm^2)/ head length (cm)	23	0.544	**0.007**
DVH of the tongue at the level of the hard palate (cm)/head length (cm)	24	0.562	**0.004**
DVH of the tongue at the level of the lingual process (cm)/head length (cm)	24	0.690	**<0.001**

Statistically significant results highlighted in bold. DVH- dorsoventral height; cm- centimetres.
