# Supplementary material for: Soft palate angle and basihyoid depth increase with tongue size and with body condition score in horses
Source: Equine Vet J. 2025 Jan 2;57(4):967–76. doi: 10.1111/evj.14445 (PMC12135754; doi:10.1111/evj.14445)
Supplement: Supplementary file 8 — Table S6. Summary of results from Mann–Whitney U test comparing all measured variables between groups of male and female. [file EVJ-57-967-s009.pdf]

Equine Veterinary Journal BEVA

Table S6. Summary of results from Mann-Whitney U test comparing all measured variables between groups of male and female.

Variable	Sample size		Median		W-value	*P*-value
	Male	Female	Male	Female		
Head length (cm)	16	8	50.053	47.880	226	0.118
Soft palate angle (˚)	27	17	144.587	142.053	685	0.063
Tongue area (cm^2)	24	17	271.813	257.327	538	0.375
DVH of the tongue at the level of the hard palate (cm)	27	17	10.427	10.293	621	0.754
DVH of the tongue at the level of the lingual process (cm)	27	17	8.141	7.897	627	0.647
Basihyoid depth (cm)	27	17	1.337	1.391	598	0.828
Head angle	27	17	170.4	170.01	374.00	0.847

DVH- dorsoventral height; cm- centimetres.
