# Supplementary material for: Soft palate angle and basihyoid depth increase with tongue size and with body condition score in horses
Source: Equine Vet J. 2025 Jan 2;57(4):967–76. doi: 10.1111/evj.14445 (PMC12135754; doi:10.1111/evj.14445)
Supplement: Supplementary file 9 — Table S7. Male and female summary statistics for all measured variables. DVH, dorsoventral height; cm, centimetres. [file EVJ-57-967-s008.pdf]

Equine Veterinary Journal BEVA

Table S7. Male and female summary statistics for all measured variables. DVH- dorsoventral height; cm- centimetres

Variable	Sex	Total Count	Mean	Standard Deviation	Minimum	Median	Maximum	Range
Head Length (cm)	Female	17	46.67	4.3	38.99	47.88	51.75	12.76
	Male	27	48.695	3.521	39.807	50.053	52.167	12.36
Soft Palate Angle (°)	Female	17	142.51	3.36	137.82	142.05	149.84	12.03
	Male	27	144.41	4.11	134.22	144.59	153.63	19.41
Tongue Area (cm^2)	Female	17	258.06	30.99	175.44	257.33	300.88	125.43
	Male	27	262.31	36.79	168.27	271.81	315.69	147.42
DVH of the Tongue at the Level of the Hard Palate (cm)	Female	17	10.235	1.005	7.812	10.293	11.688	3.876
	Male	27	10.326	0.868	8.149	10.427	11.826	3.677
DVH of the Tongue at the Level of the Lingual Process (cm)	Female	17	7.795	1.109	5.492	7.896	9.37	3.877
	Male	27	7.903	1.231	4.113	8.14	10.28	6.167
Basihyoid Depth (cm)	Female	17	1.4946	0.3831	0.9465	1.3905	2.3435	1.397
	Male	27	1.5057	0.4185	0.933	1.337	2.7085	1.7755

A table showing body condition score and signalment of each patient including: age, breed and sex. BCS- body condition score; y- years; m- months; X- crossbreed.
