# Supplementary material for: Soft palate angle and basihyoid depth increase with tongue size and with body condition score in horses
Source: Equine Vet J. 2025 Jan 2;57(4):967–76. doi: 10.1111/evj.14445 (PMC12135754; doi:10.1111/evj.14445)
Supplement: Supplementary file 10 — Table S8. Results of Spearman's rank correlation between age and all computed tomography measured variables. [file EVJ-57-967-s002.pdf]

Equine Veterinary Journal BEVA

Table S8. Results of Spearman's rank correlation between age and all computed tomography measured variable.

**Variable**	**Number of values**	**Spearman's r**	***P*-value**
Head length (cm)	24	-0.071	0.741
Soft palate angle (˚)	44	0.105	0.499
Tongue area (cm^2)	41	-0.009	0.953
DVH of the tongue at the level of the hard palate (cm)	44	0.110	0.476
DVH of the tongue at the level of the lingual process (cm)	44	0.180	0.243
Basihyoid depth (cm)	44	0.147	0.343
Head angle (˚)	44	-0.324	**0.032**

DVH- dorsoventral height; cm- centimetres. Stastically significant results highlighted in bold.
